# A Rare Case Report of Intraventricular Anaplastic Pleomorphic Xanthoastrocytoma

**DOI:** 10.7759/cureus.35975

**Published:** 2023-03-10

**Authors:** Sofia Bettencourt, Gonçalo Almeida, Tiago Maia

**Affiliations:** 1 Neuroradiology, Centro Hospitalar Lisboa Central, Lisbon, PRT; 2 Pathology, Instituto Português de Oncologia de Lisboa Francisco Gentil, Lisbon, PRT

**Keywords:** braf, epithelioid glioblastoma, intraventricular tumor, anaplastic, pleomorphic xanthoastrocytoma

## Abstract

We describe a rare case of a 33-year-old man presenting with a three-day history of dizziness and memory impairment. On clinical examination, he had a wide-based gait and postural instability. Laboratory tests were unremarkable. The patient underwent a CT scan, which showed an intraventricular heterogeneous mass, with calcifications. An MRI scan was performed, revealing a well-defined intraventricular lesion, with cystic and necrotic areas, hemorrhagic components, areas of restricted diffusion, and a peripheral solid component with post-contrast enhancement. This lesion was ultimately diagnosed as an anaplastic form of pleomorphic xanthoastrocytoma (PXA) (WHO grade 3).

Prototypical PXA is a rare low-grade astrocytic tumor, almost always hemispheric. To our knowledge, this is only the third case report to describe an intraventricular PXA. Anaplastic forms of PXA have a more aggressive behavior and should be distinguished from other high-grade astrocytic neoplasms, especially from glioblastoma, isocitrate dehydrogenase (IDH)-wildtype variants (GB). Histopathological features of anaplastic forms of PXA (WHO grade 3) with epithelioid features are very similar to those of epithelioid glioblastoma and its differentiation is a common diagnostic challenge that should prompt genetic testing. Distinguishing between these two entities is crucial since the former is associated with significantly more survival benefits from targeted therapies (MAPK pathway inhibitors).

## Introduction

Pleomorphic xanthoastrocytoma (PXA) accounts for less than 1% of all astrocytic neoplasms and usually affects children and young adults presenting with epilepsy [1,2]. Prototypical PXA is hemispheric, low-grade, well-circumscribed tumors with a distinct morphology characterized by the cohesive proliferation of pleomorphic astrocytes with pericellular reticulin network and a paradoxically very low mitotic activity correlating with indolent clinical evolution (WHO grade 2). Anaplastic forms of PXA (A-PXA) (WHO grade 3) are more aggressive but should be distinguished from other high-grade astrocytic neoplasms, especially from glioblastoma, isocitrate dehydrogenase (IDH)-wildtype variants (GB). This distinction, especially in de novo A-PXA, may be difficult and cause diagnostic pitfalls, which may be avoided by genetic studies since PXA (and GB) harbor typical recurrent genetic alterations. Imaging and intraoperative findings are valuable in disclosing the well-circumscribed nature of the neoplasm, in contrast with diffuse gliomas, although they are of limited value in the differential diagnosis of A-PXA with epithelioid and giant cell variants of GB. This distinction is relevant not only for proper prognostication but also, most importantly, because patients with A-PXA benefit from targeted therapy directed to the constitutional MAPK pathway activation that characterizes the biology of these tumors. Although almost always hemispheric, the hypothesis of A-PXA should be considered when encountering tumors arising in other locations as well. In this report, we present a rare case of an intraventricular A-PXA. To our knowledge, this is only the third case report to describe a PXA involving the ventricular system [3,4].

## Case presentation

A 33-year-old man, with no relevant personal or family medical history, presented with a three-day history of dizziness and memory impairment. He had no previous history of seizures and denied abnormal motor movements, loss of consciousness, or urinary incontinence. On clinical examination, he had a wide-based gait and postural instability. Laboratory tests were unremarkable.

**Figure 1:**
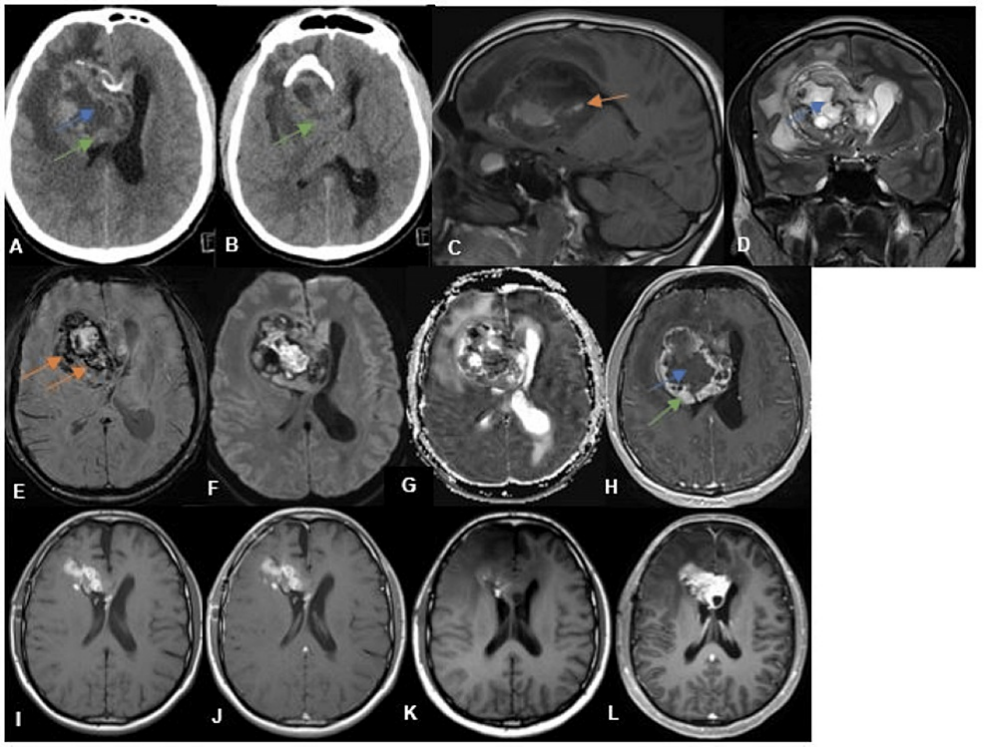
CT and MRI findings (A, B) CT scan showed an apparently intraventricular mass lesion centered in the right lateral ventricle. Brain MRI sagittal T1 (C), coronal T2 (D), SWI (E), DWI (F), ADC map (G), and T1 post-contrast (H) showed a heterogeneous right intraventricular mass lesion expanding the frontal horn and body. The lesion was mostly hypointense on T1, hyperintense on T2 with cystic and necrotic areas (blue arrows), hemorrhagic components (orange arrows), and a peripheral solid component (green arrows), with areas of restricted diffusion and post-contrast enhancement of the solid component. Additionally, perilesional edema and hydrocephalus were observed. Post-surgery MRI axial T1 (I) and T1 post-contrast (J) showed tumor resection and post-surgery subacute blood products. Brain MRI axial T1 (K) and T1 post-contrast (L) seven months after surgery demonstrated tumor recurrence CT: computed tomography; MRI: magnetic resonance imaging

The patient underwent a CT scan, which showed an apparently intraventricular heterogeneous mass lesion, with calcifications (Figures 1A, 1B). Further investigation with an MRI scan (Figures 1C-1H) revealed a right intraventricular mass lesion, with well-defined borders, measuring 63 x 59 x 54 mm (anteroposterior x transverse x vertical), expanding the right frontal horn and body. It demonstrated cystic and necrotic areas, hemorrhagic components, areas of restricted diffusion, and a peripheral solid component with post-contrast enhancement. Additionally, perilesional edema and hydrocephalus were observed, causing sulcal effacement. The imaging features suggested a neoplastic lesion. The differential diagnosis of this heterogeneous intraventricular mass lesion mainly included central neurocytoma, ependymoma, and glioblastoma.

**Figure 2:**
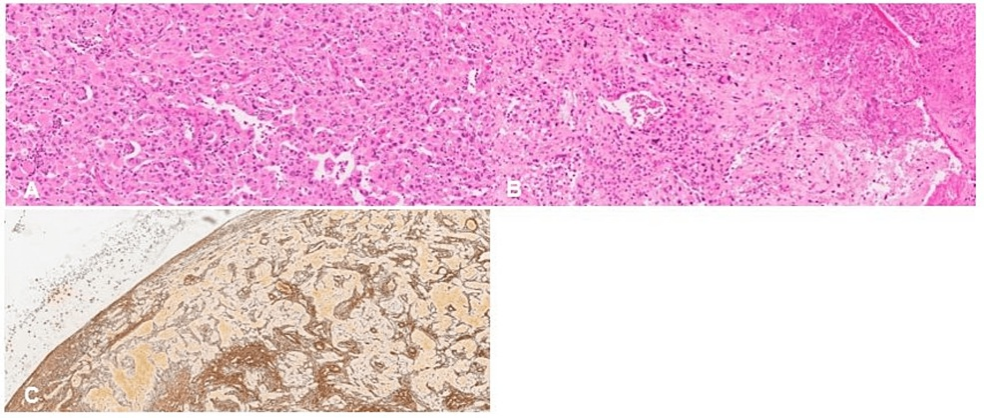
Histological features (A) H&E, medium power: highly cellular/cohesive neoplasm composed of pleomorphic epithelioid/rhabdoid astrocytes with abundant dense pink cytoplasm. Some small lymphocytic infiltrates permeate between tumor cells. (B) H&E, medium power: areas of tumoral necrosis and plump endothelial cells without overt microvascular proliferation. (C) Gomori stain, medium power: tumoral reticulin network also highlights the non-infiltrative nature of the neoplasm. Some larger areas are devoid of reticulin network

Surgical tumor resection revealed a slightly hemorrhagic intraventricular mass lesion, with well-defined borders. Histologic examination showed a well-circumscribed compact tumor composed of large pleomorphic epithelioid to rhabdoid astrocytic cells with abundant eosinophilic cytoplasm, sometimes multinucleated (Figure 2A), with significant mitotic activity (more than five mitoses per 10 high-power field). Incipient microvascular proliferation and necrosis were present (Figure 2B). Perivascular and pericellular small lymphocytes were also present. The tumor exhibited an abundant underlying reticulin network, including at the periphery, enclosing individual or small groups of neoplastic cells. Some areas devoid of reticulin were present and associated with necrotic foci, consistent with the high-grade progression of a previous low-grade neoplasm (Figure 2C). No xanthomatous change or eosinophilic granular bodies were identified.

The tumor cells exhibited the following immunoprofile: GFAP+, OLIG2+, IDH1 R132H-, ATRX+, INI1+, BRG1/SMARCA4+, and H3K27me3+ and H3K27M-, confirming the astrocytic nature of the neoplasm and excluding the possibilities of astrocytoma, IDH-mutant, midline diffuse glioma H3K27-altered and atypical teratoid/rhabdoid tumor.

At this point, the diagnoses of PXA (WHO grade 3), and epithelioid glioblastoma (EGB), a variant of glioblastoma, IDH-wildtype (GB) (WHO grade 4) were considered. Another variant of GB, giant cell glioblastoma (GCGB), was excluded based on morphologic criteria (only scattered multinucleated tumor cells with no syncytial arrangement). These entities share the features of good circumscription and the presence of voluminous epithelioid cells, as present in this case.

Wide genetic testing was performed in an attempt to better classify the neoplasm, which revealed the following alterations: BRAFV600E mutation (PCR), CDKN2A homozygous deletion (FISH), TERT promotor mutation (C228T) (PCR), and complex karyotype (CGH) in the absence of whole 7/10 chromosome gain/loss respectively (CGH), EGFR amplification (FISH), IDH1/2 mutation (PCR), or H3F3A mutation (PCR), typical genetic signatures of PXA. A final diagnosis of PXA (WHO grade 3) was made.

Macroscopic complete resection of the lesion was achieved (Figures 1I, 1J). The patient received adjuvant therapy, with combined radiotherapy (60 Gy) and chemotherapy with temozolomide. Two years have passed since the surgery. The patient is alive, but he was lost to follow-up at the end of the first year.

Following the third cycle of chemoradiotherapy, the patient presented with episodic confusion and aphasia. MRI scan showed local tumor recurrence (Figures 1K, 1L). He then started treatment with dabrafenib (150 mg twice daily) and trametinib (2 mg) with clinical improvement. A follow-up MRI was scheduled to be performed one month after the start of the treatment, and then every three months thereafter; however, the patient was then lost to follow-up.

## Discussion

Intraventricular tumors are rare entities, accounting for 0.8-1.6% of all intracranial tumors. They are mostly benign and can be classified into primary tumors, which derive from the ependymal and subependymal lining, choroid plexus, and septum pellucidum. Most metastatic lesions in this region arise from the choroid plexus due to its high vascularity. Secondary tumors involve the invasion of the ventricular region by adjacent brain tumors [4].

PXA is a rare low-grade astrocytic tumor. It was first described in 1979 by Kepes and colleagues and was included in the WHO classification of tumors of the central nervous system (CNS) in 1993. PXA has been classified as a WHO grade 2 tumor. However, 15-50% of these tumors have shown areas of necrosis, increased vascular proliferation, and mitotic activity (defined as five or more mitoses in 10 high-power fields). In line with these changes, in 2007, the WHO classification of CNS tumors acknowledged the subgroup of “PXA with anaplastic features” pertaining to lesions with more aggressive clinical behavior and histologic features. In 2016, the new classification of anaplastic pleomorphic xanthoastrocytomas (A-PXA) was added, and in the fifth edition of the WHO classification of CNS tumors, the designation of these two entities was changed to CNS WHO grade 2 and 3 (previously A-PXA) [1,2,3].

PXA usually occurs in children and young adults, and the mean age at diagnosis is 26.3 years. It affects male and female patients equally. Symptoms at onset include headaches, focal neurological symptoms, and epileptic seizures [1,4,5]. Neuroimaging frequently demonstrates a cystic lesion with a mural nodule. It is thought to originate from subpial astrocytes, indicating its frequent superficial location in the cerebral hemispheres, particularly in the temporal lobe [5]. Less frequently described locations include the cerebellum, spinal cord, sellar region, and retina [1]. To our knowledge, our case is only the third case of an intraventricular CNS WHO grade 3 PXA to be reported in the literature [3,4].

Imaging features of these lesions are similar to those of high-grade astrocytomas, including large, heterogeneous-appearing masses, hypointense on T1 and hyperintense on T2, with prominent peritumoral edema. Intense heterogeneous contrast enhancement is typical, sometimes accompanied by a “dural tail” sign. The increased vascularity of these tumors and the presence of necrosis and hemorrhage could help to explain its enhancement pattern [1,6]. Characteristic histopathological features of these lesions closely resemble those seen in rare variants of glioblastoma, such as EGB and GCGB, particularly when epithelioid features are present [1,4,7].

EGB also shares some molecular features with grade 3 PXA, including the high incidence of BRAF V600E mutation, making its distinction a common challenge [7,8]. Molecularly, our tumor exhibited the “PXA signature” (CDKN2A homozygous deletion, BRAF mutation, TERT promotor mutation) without the more specific molecular defining features of GB (absence of whole 7/10 chromosome gain/loss respectively and absence of EGFR amplification). BRAF mutation, scarcity of both multinucleated cells and of more abundant bizarre/pleomorphic cells tend to exclude GCGB [1,7].

The current WHO classification (5th edition) recognizes both morphologic and genetic overlap between some EGB and PXA with no solid criteria to distinguish between the two, especially in the absence of a previous history of histologically diagnosed PXA (WHO grade 2). Nevertheless, a dense reticulin network was not to be expected in EGB and the whole constellation of the findings led us to favor a diagnosis of grade 3 PXA over EGB.

CNS WHO grade 2 PXA has a good prognosis, with a 10-year survival of over 70%; however, it can recur and progress, and hence early surgical complete resection is crucial. Malignant transformation (PXA WHO grade 3/A-PXA) is seen in 10-20% of the cases, in a period ranging from seven months to 15 years [5]. After gross total resection, adjuvant therapy (including radiotherapy and possibly chemotherapy) and close surveillance are needed in cases of CNS WHO grade 3 PXA [1]. A-PXA may result from a progression of PXA (WHO grade 2) or have a de novo presentation. A poor prognosis has been associated with this entity in the literature, with a five-year survival of 57.1% [6]. BRAF V600E mutations, responsible for constitutively activating the RAS/RAF/MEK/ERK signaling pathway, are found in 38-60% of patients with PXA, particularly in 17-65% of grade 3 PXA, and in 50% of epitheloid glioblastoma [9,10,11]. The introduction of BRAF inhibitor therapies (such as dabrafenib and trametinib) in patients with BRAFV600E mutant gliomas has shown favorable clinical and radiographic responses, with a durable antitumor activity [9,12,13]. Nonetheless, their efficacy seems to vary according to histologic subtype. A radiographic response or stabilization was achieved in more than 50% of patients with BRAFV600E mutant PXA [14]. Moreover, an objective response rate for patients with BRAF V600 mutant PXA treated with vemurafenib monotherapy was found in 42.9%, in contrast to 9.1% for patients with glioblastoma IDH-wildtype subgroup [14]. Overall, published case reports demonstrate up to 35 months of stable disease when BRAF and MEK inhibitor treatment regimens are used to treat BRAFV600E-mutated PXA [15].

## Conclusions

This case report emphasizes that PXA (WHO grade 3), although rare, should be included in the differential diagnosis of intraventricular lesions. Key demographic and imaging findings may help narrow down the differential diagnosis, namely the typical well-circumscribed borders, which are frequent in PXA lesions, and the extensive surrounding edema and central hypointensity suggestive of necrosis, suggestive of an anaplastic type. Nonetheless, a definitive diagnosis warrants histological examination, immunohistochemical analysis, and genetic testing.
